# The roles of toll-like receptor 4, CD33, CD68, CD69, or CD147/EMMPRIN for monocyte activation by the DAMP S100A8/S100A9

**DOI:** 10.3389/fimmu.2023.1110185

**Published:** 2023-03-28

**Authors:** Alexander Möller, Saskia-Larissa Jauch-Speer, Shrey Gandhi, Thomas Vogl, Johannes Roth, Olesja Fehler

**Affiliations:** Institute of Immunology, University of Münster, Münster, Germany

**Keywords:** S100A8/A9, CRISPR, monocytes, DAMP, TLR 4 receptor

## Abstract

The S100A8/A9 heterocomplex is an abundant damage-associated molecular pattern and mainly expressed by monocytes, inflammatory activated keratinocytes and neutrophilic granulocytes. The heterocomplex as well as the heterotetramer are involved in a variety of diseases and tumorous processes. However, their detailed mode of action and especially which receptors are involved hereby remains to be fully revealed. Several cell surface receptors are reported to interact with S100A8 and/or S100A9, the best studied being the pattern recognition receptor TLR4. RAGE, CD33, CD68, CD69, and CD147, all of them are involved as receptors in various inflammatory processes, are also among these putative binding partners for S100A8 and S100A9. Interactions between S100 proteins and these receptors described so far come from a wide variety of cell culture systems but their biological relevance *in vivo* for the inflammatory response of myeloid immune cells is not yet clear. In this study, we compared the effect of CRISPR/Cas9 mediated targeted deletion of CD33, CD68, CD69, and CD147 in ER-Hoxb8 monocytes on S100A8 or S100A9 induced cytokine release with TLR4 knockout monocytes. Whereas deletion of TLR4 abolished the S100-induced inflammatory response in monocyte stimulation experiments with both S100A8 and S100A9, knockouts of CD33, CD68, CD69, or CD147 revealed no effect on the cytokine response in monocytes. Thus, TLR4 is the dominant receptor for S100-triggered inflammatory activation of monocytes.

## Introduction

The innate immune system reacts as a first defense mechanism immediately in response to invading pathogens or tissue damage. A crucial cellular mechanism is the recognition of PAMPs (pathogen-associated molecular patterns) *via* PRRs (pattern recognition receptors) on cells of the innate immune system, which induces activation of these cells resulting in cytokine and chemokine release. Well-known groups of these PRRs include scavenger receptors, C-type lectin receptors, and toll-like receptors (TLRs) (1–3). The innate immune response can also be triggered by DAMPs (damage-associated molecular patterns), mostly intracellularly stored proteins secreted in the context of inflammation or tissue damage (4). Prominent members of this group of molecules during many inflammatory diseases are the proinflammatory proteins S100A8 (myeloid-related protein-8, Mrp8) and S100A9 (myeloid-related protein-14, Mrp14), two members of the calcium-binding S100 family that are expressed primarily by neutrophils and monocytes (5). S100A8 and S100A9 are highly abundant DAMPs in many clinically relevant diseases such as autoimmune diseases, allergies, cancer, cardiovascular diseases or infections and also participate in cytoskeletal rearrangement (6).

Recently we could show that the relevant *in vivo* form of S100A8 and S100A9 are heterodimers (7). Excess of released heterodimers inactivates itself by tetramer formation restricting the inflammatory response of this DAMP locally. Heterodimers as well as heterotetramers of S100A8 and S100A9 are also known as calprotectin.

Their inflammatory activity is of functional relevance in arthritis, psoriasis, contact dermatitis, infections, cardiovascular diseases or tumor immunity and metastasis (7–14). One mechanism of the proinflammatory activity of these proteins is based on the interaction of S100A8/A9 complexes with TLR4 expressing cells, which causes the release of various cytokines, e.g. interleukin 6 (IL-6) or TNF-α (15).

However, several other receptors have been described as binding partners for S100A8 and S100A9, including RAGE, CD33, CD68, CD69, and CD147 (16–19).

CD33, also known as Siglec-3, is mainly expressed on myeloid progenitor cells and belongs to the Siglec (sialic acid-binding Ig superfamily lectin) family. Siglecs, which are characterized by sequence similarities and the ability to bind sialic acid, are associated with features of inhibitory receptors. The inhibitory function of CD33 is related to its cytosolic immunoreceptor tyrosine-based inhibitory motif (ITIM) and recruitment of protein phosphatases such as SHP-1 (20–22). In addition to sialic acid, S100A9 has been described as a ligand for CD33 that can activate the receptor on human bone marrow cells and induce the release of IL-10 and TGF-ß (16).

CD68 and its murine ortholog macrosialin are mainly expressed by macrophages and can be assigned to the lamp/lgp family, a family of lysosomal membrane associated glycoproteins (23). The function of CD68 is poorly understood, but due to its ability to bind oxLDL (oxidated low-density lipoprotein), a possible role as a scavenger receptor is hypothesized (24). Other ligands besides oxLDL include phosphatidylserine and possibly S100A8 and S100A9 (25). It has been shown in rat macrophages that both S100A8 and S100A9 bind to CD68 and that their interaction could influence TNF-α and IL-6 release in addition to an autocrine enhanced release of S100 proteins (19).

CD69 is a transmembrane glycoreceptor that belongs to the C-type lectin receptor family and is described as an early activation marker by many hematopoietic leukocyte cells (26, 27). CD69 is known for its involvement in the differentiation of regulatory T cells *via* the Jak3/Stat5 signaling pathway (28). Gal-1 has been identified as a specific ligand for CD69, but the S100A8/S100A9 complex is also a putative ligand that was found to influence T cell differentiation as well as the regulation of cytokines such as IL-17A and TGF-ß (18, 29, 30).

CD147 or EMMPRIN (extracellular matrix metalloproteinase inducer) is a transmembrane glycoprotein that belongs to the immunoglobulin superfamily (31).

Particularly high expression levels are associated with a wide variety of tumorous processes (32, 33). Many different proteins have been associated with EMMPRIN as stimulants, most notably cyclophylins (34). Using affinity isolation-mass spectrometry, it was shown that S100A8 binds to EMMPRIN. There is evidence that S100A9 induces cytokines and matrix metalloproteases (MMPs) in melanoma cells *via* binding to EMMPRIN (17).

RAGE (receptor for advanced glycation end products) is also frequently described as a receptor for both S100A8 and S100A9 that transduces its signal *via* the p38 MAPK/NF-kB pathway (35, 36). However, we already demonstrated the subordinate role of RAGE as an S100 receptor inducing inflammatory activation (15), so this receptor was not included again in the study.

In this study, we aimed to confirm or refute the relevance of interactions of S100A8/A9 with the receptors CD33, CD68, CD69, and CD147 regarding inflammatory activation of monocytes. We used monocytic ER-Hoxb8-derived cells and the CRISPR/Cas9 gene editing system. These murine myeloid progenitor cells are transiently immortalized by estrogen-regulated (ER) expression of the Hoxb8 transcription factor which blocks progenitor differentiation resulting in stably growing myeloid progenitor cells. After withdrawal of estrogen the cells retain the potential to differentiate into monocytes which behave phenotypically and functionally like their primary equivalents, stop proliferation and show a typical life span in culture while maintaining their regular innate immune function (37). CRISPR/Cas9 was used to generate knockout cell lines for each of the receptors CD33, CD68, CD69, and CD147. By stimulating all cell lines with the S100A8 and S100A9 followed by multiplex cytokine analysis, we aimed to compare receptor-specific effects on inflammatory activation of monocytes.

## Materials and methods

### Cell culture

ER-Hoxb8 cells were prepared as described previously (37) and cultivated in RPMI medium (Thermo Fisher Scientific) with 10% FBS (Biowest), 1% glutamine (Thermo Fisher Scientific), 1% penicillin/streptomycin (Sigma-Aldrich), 40 ng/ml rmGM-CSF (ImmunoTools) and 1 µM β-estradiol (Sigma-Aldrich). For differentiation, progenitor cells were incubated in this medium without the added ß-estradiol for a period of five days. DMEM (Thermo Fisher Scientific) was used as HEK293T cell culture medium, supplemented with 10% FBS (Biowest), 1% glutamine (Thermo Fisher Scientific), 1% penicillin/streptomycin (Sigma-Aldrich) and 1% sodium pyruvate (Merck). Cultivation of all cell lines was performed at 37°C and 5% CO_2_.

### Cell generation

Cas9-expressing myeloid cells were obtained from Cas9-expressing mice and ER-Hoxb8 cells were prepared in the presence of GM-CSF to obtain so called “GM-CSF-ER-HoxB8 progenitors” which are committed to monocyte-macrophage differentiation (37). CD33, CD68, CD69, CD147 and TLR4 KO ER-Hoxb8 cells were generated using the CRISPR/Cas9 gene editing system as described previously (38). The sequences of the gRNAs used can be found in **Supplementary Table 1**. For lentivirus production, lentiGuide-Puro-gRNA, the envelope expressing plasmid pCMV-VSV-G (AddGene, #8454) and the packaging plasmid psPAX2 (AddGene, #12260) were co-transfected in HEK293T cells. For transduction, lentiviral particles and 8 µg/ml polybrene (Sigma-Aldrich) were added to the ER-Hoxb8 cells, followed by a one-hour spinfection with subsequent change of medium. Successfully transduced cells were selected with increasing amounts of puromycin (*In vivo* Gen).

### Detection of mutations caused by CRISPR/Cas9

Genomic DNA of ER-Hoxb8 cells was isolated using the QIAamp^®^ DNA Blood Mini Kit (QIAGEN) according to the manufacturer’s instruction. Target DNA regions were amplified by PCR using specific primers for each target (listed in **Supplementary Table 2**), dNTPs, Q5 reaction buffer (Thermo Scientific) and Q5 High-Fidelity DNA Polymerase (Thermo Scientific). Amplicons were separated on an agarose gel and then selectively excised. NucleoSpin^®^ Gel and PCR clean-up Kit (Macherey‐Nagel) and CloneJET PCR Cloning Kit (Thermo Scientific) were used according to the manufacturer’s instruction to purify PCR-products and clone them in a pJET vector. Plasmid DNA was transformed into competent DH5α cells (Invitrogen), which were seeded on agar plates with LB-medium containing 100 mg/L ampicillin (Sigma), selecting for DH5α bacteria carrying pJET plasmids. After incubation overnight at 37°C, single colonies were inoculated in LB-medium with 100 mg/L ampicillin and incubated again overnight at 37°C for subsequent mini-culture preparation. After RNA degradation with RNAse A (Sigma), DNA was isolated after lysis with SDS and NaOH, precipitation with isopropanol and washing with 70% ethanol, DNA pellets were completely dried and resuspended in ddH_2_O. Barcode Economy Run Service of Seqlab Sequence Laboratories (Göttingen, DE) was used for sequencing.

### Cell stimulation

Differentiated cells were counted and incubated in estradiol-free cell culture medium either without addition, with 10 ng/ml LPS, with 5 µg/ml S100A8 homodimers or with 10 µg/ml S100A9 homodimers for 4 hours at 37°C, 5% CO_2_ (S100A8 and S100A9 preparations originated from our own production). For the stimulation experiments S100-homodimers were used instead of the heterodimer S100A8/A9 because of the rapid tetramer formation in calcium ion containing medium. S100A8/A9 tetramers are unable to bind to TLR4/MD2 due to that the interaction site of S100A8/A9 is hidden in the tetramer interface. After centrifugation, cell culture supernatants were collected and frozen directly at -80°C until used for further analysis.

### Measurements of TNF-α and IL-6 protein levels

TNF-α and IL-6 in cell culture supernatants were quantified according to the manufacturer’s instruction using the BioLegend LEGENDplex™ Mouse Macrophage/Microglia panel (Cat No. 740846, Lot B305067). The absolute concentrations of TNF-α and IL-6 were determined using LEGENDplex™ v8.0 data analysis software based on standard curves recorded for each run.

### Flow *cytometry*

Cells were washed and counted before the addition of the coupled antibodies or corresponding isotype controls listed in **Supplementary Table 3**. After incubation in the dark, cells were washed again, resuspended in PBS containing 1% FBS and measured by flow cytometry (Navios, Beckmann Coulter). Data was analyzed using FlowJo v10 (Treestar Inc.).

### Statistics

Prism 5.0 software (GraphPad Software, CA, USA) was used to determine the statistical significance of the data. Comparisons among multiple groups were performed by using one-way ANOVA, followed by Dunnett’s test for identifying significant deviations from a control group. To assess the influence of more than one variable on a target variable, a two-way ANOVA was applied, followed by Dunnett’s test for identifying significant deviations from a control group. Differences were considered statistically significant at a probability (p‐value) of ≤0.05.

## Results

### CD33, CD68, CD69, CD147 and TLR4 knockout using CRISPR/Cas9

**Figure 1:**
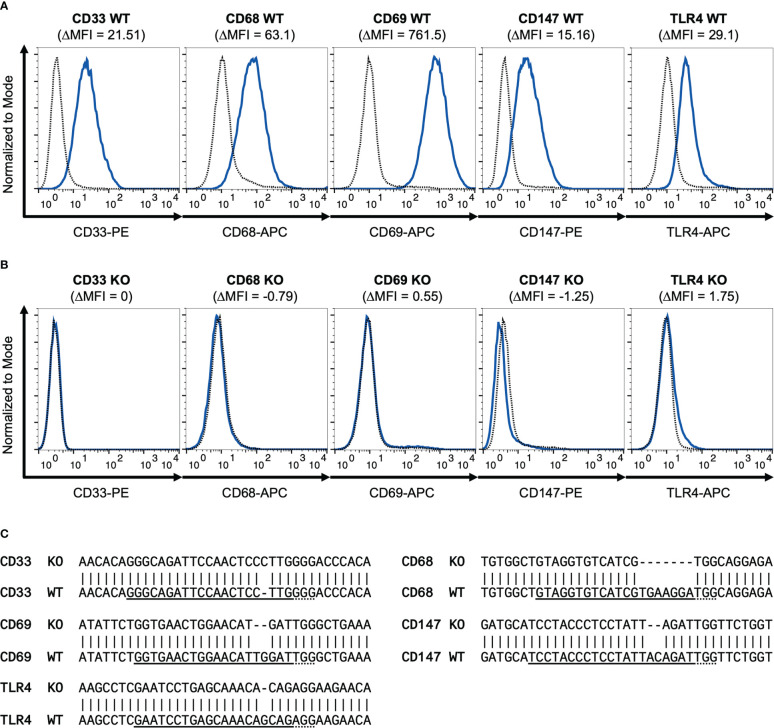
Validation of CD33, CD68, CD69, CD147 and TLR4 knockout. Flow cytometry histograms show WT **(A)** and different CRISPR/Cas9 knockout **(B)** ER-Hoxb8 precursor cells stained for surface CD33, CD68, CD69, CD147 and TLR4. Dotted lines represent corresponding isotype controls. Delta MFI values are calculated as differences of geometric mean fluorescence intensities. **(C)** Representative sequencing results of PCR amplicons of genomic target regions cloned into pJET-vector are shown for knockout cell lines examined and compared with wild-type gene sequences. gRNA sequences are underlined. Dotted lines represent PAM sequences. Mismatches indicate mutations.

To better understand the relevance of the receptors CD33, CD68, CD69 and CD147 compared to TLR4 in S100A8 and S100A9 mediated inflammation, Cas9 expressing ER-Hoxb8 precursor cells (hereinafter referred as ´wild-type´) were used to generate a knockout cell line for each receptor. In addition, TLR4 knockout cells were generated from the same starting cells, which served as controls in subsequent experiments. All five receptors could be detected on the non-differentiated ER-Hoxb8 wild-type cells by flow cytometry (**Figure 1A**). The same protocol was then used to validate the individual CRISPR/Cas9 knockouts (**Figure 1B**). Additionally, the genomic sequence of single cells was analyzed by DNA isolation, bacterial transformation and subsequent plasmid sequencing to reveal mutations in the region of the guideRNAs used. All sequences examined here showed mutations that lead to a shift in the reading frame and thus to a gene knockout (**Figure 1C**).

### Knockout macrophages vary in their expression of certain surface markers but showed no general block of differentiation

**Figure 2:**
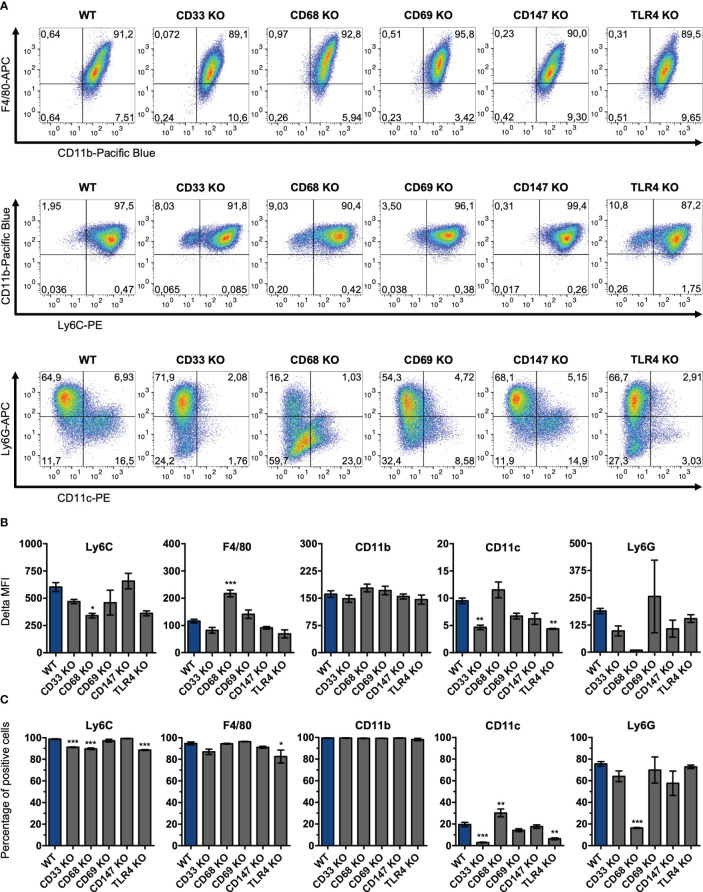
Surface expression levels of differentiation markers on knockout ER-Hoxb8 macrophages. CD33, CD68, CD69, CD147 and TLR4 knockout ER-Hoxb8 precursor cells, as well as WT ER-Hoxb8 precursor cells were differentiated into macrophages and subsequently analyzed by flow cytometry (n = 3) regarding their expression of Ly6C, F4/80, CD11b, CD11c and Ly6G. Representative pseudocolor plots **(A)**, Delta MFI values **(B)** and percentages of marker positive cells **(C)** are shown for each cell line. Delta MFI values are calculated as differences of geometric mean fluorescence intensities. Values are the means ± SEM. *p < 0.05, **p < 0.01, ***p < 0.001, by one-way ANOVA and Dunnett's test in comparison to WT.

For stimulation experiments, ER-Hoxb8 wild-type cells and the five different knockout cell lines were differentiated into macrophages over five days with GM-CSF. These differentiated cells were also examined for their expression of the monocytic differentiation markers Ly6C, F4/80, CD11b, CD11c, and Ly6G *via* flow cytometry to determine whether the absence of any of the receptors affected their differentiation behavior (**Figure 2**). At the qualitative level, cells appeared to exhibit a similar differentiation pattern, which can best be summarized as F4/80^hi^CD11b^hi^Ly6C^hi^Ly6G^+^CD11c^-/lo^ (**Figure 2A**). The only exception was the CD68 KO cell line with visibly reduced Ly6G expression. In detail, however, some quantitative differences could be identified by comparing MFI values (**Figure 2B**). We observed that CD68 knockout cells expressed significantly less Ly6C than wild-type cells *(WT* vs. *CD68 knockout, p=0.034)* and also higher levels of F4/80 (WT vs. CD68 knockout, p=0.0002). Both the CD33 knockout cells and TLR4 knockout cells showed significantly lower expression of CD11c than the wild-type cell line (WT vs. CD33 knockout, p=0.004; WT vs. TLR4 knockout, p=0.003). Some further minor but non-significant deviations were observed for Ly6G. CD11b expression was approximately comparable between all groups. Thus, the receptor knockouts appear to affect the differentiation behavior of cells in different ways, which may also have implications for the inflammatory potency of individual cell lines. However, comparison of the percentages of marker-positive cells (**Figure 2C**) confirms the visual impression of the dot plots that Ly6G in CD68 KO cells is the only differentiation marker with a strong deviation in expression compared with wild-type cells in this study. None of the knock-outs induced a general block of monocyte differentiation.

### CD33, CD68, CD69 and CD147 are not involved in ER-Hoxb8 macrophage activation induced by S100A8 or S100A9.

**Figure 3:**
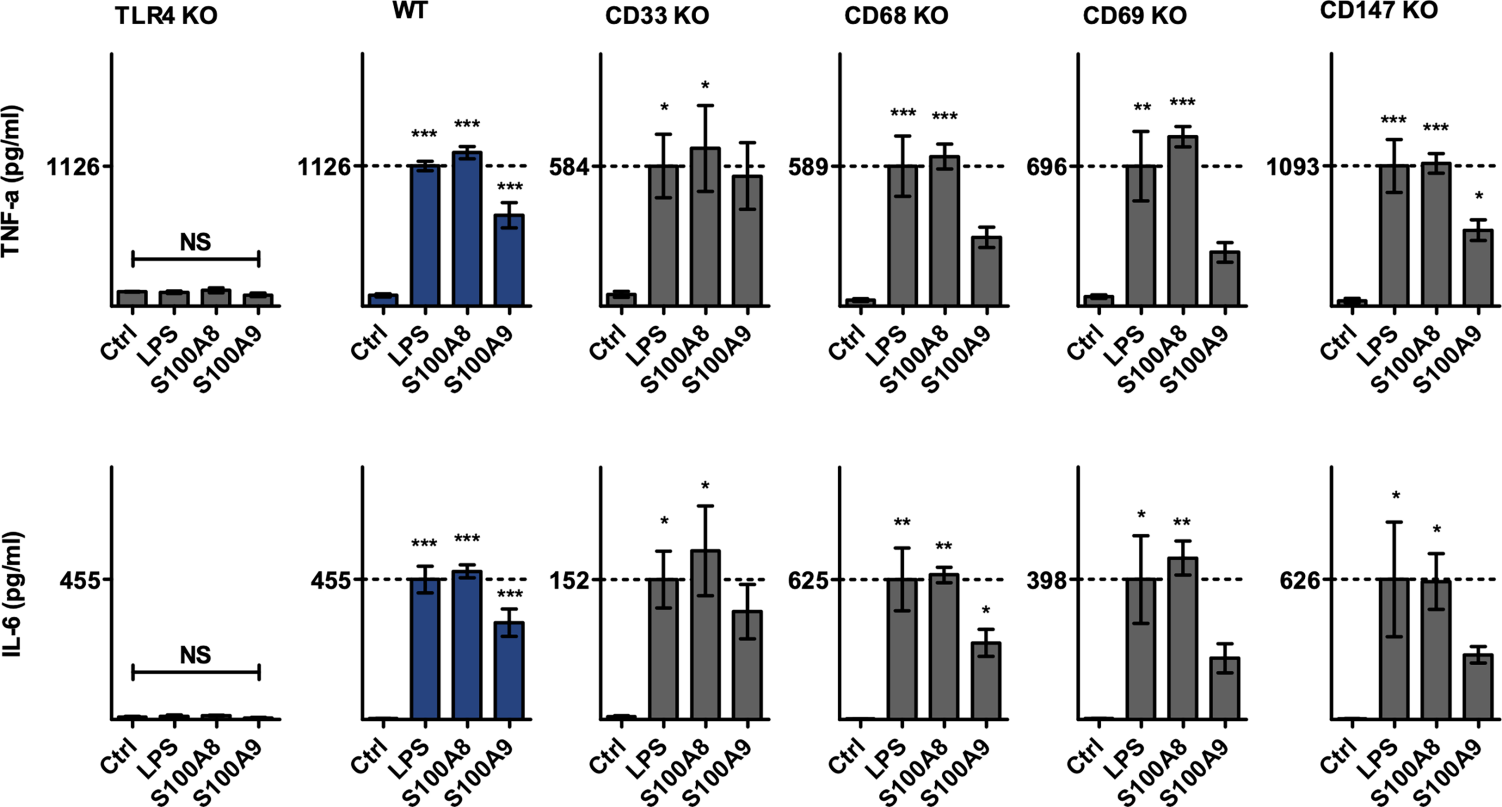
TNF-α and IL-6 upregulation upon S100A8 and S100A9 stimulation. CD33, CD68, CD69, CD147 and TLR4 knockout ER-Hoxb8 precursor cells, as well as WT ER-Hoxb8 precursor cells were differentiated into macrophages and subsequently stimulated with LPS, S100A8 homodimer, S100A9 homodimer or incubated without stimulus (Ctrl). TNF-α and IL-6 protein levels in cell culture supernatants were quantified using LegendPlex™ assay (n = 3). Concentrations below the limit of quantification were set as LLOQ/2. Values are the means ± SEM. *p < 0.05, **p < 0.01, ***p < 0.001, NS = not significant, by one-way ANOVA and Dunnett's test in comparison to Ctrl.

To investigate the interactions of S100A8 and S100A9 with the different potential S100 receptors, the differentiated macrophages were stimulated either with S100A8, S100A9 or with LPS. For the stimulation experiments S100-homodimers were used instead of the heterodimer S100A8/A9 because of the rapid tetramer formation in calcium ion containing medium. Tetramers of S100A8/A9 are inactive in terms of inducing an inflammatory response (7). Subsequently, cell culture supernatants were analyzed regarding released cytokines using the multiplex assay LEGENDplex™. Supernatants were analyzed for the presence of interleukins IL-18, IL-23, IL-10, IL-12p70, IL-6, and IL-12p40, as well as TGF-ß and TNF-α. Upon stimulation of ER-Hoxb8 macrophages with LPS, S100A8, or S100A9, detectable levels were measured only for IL-6 and TNF-α (**Figure 3**). The levels of the other cytokines mentioned were below the limit of quantification.

Due to the variability of differentiation of the different knockout cells shown above, the cytokine release in response to LPS stimulation as the natural ligand of TLR4 activation showed some quantitative differences. As expected, TLR4 knockout cells did not secrete TNF-α or IL-6 under LPS, S100A8 or S100A9 stimulation, whereas differentiated wild-type cells exhibited significant detectable amounts of both TNF-α and IL-6 in their supernatant when incubated with either LPS, S100A8 or S100A9. In contrast to knockout of TLR4, knockouts of CD33, CD68, CD69, or CD147 showed a clear induction of TNF-α and IL-6 in response to S100A8 stimulation which was in a similar range as observed for LPS treatment. S100A9 showed also a clear induction of TNF-α and IL-6 in wildtype cells and knockouts of CD33, CD68, CD69, or CD147 although at a lower level compared to LPS and S100A8. The ratio of cytokines released under S100A8 and S100A9 stimulation relative to the amounts released by LPS was of comparable value between wild-type cells and these four knockout cell lines examined. Statistical analysis revealed no significant differences between these cell lines in terms of the relative amounts of cytokines released (**Supplementary Figure S1**).This leads to the assumption that in the investigated model of ER-Hoxb8 macrophages there is no receptor-specific interaction of the proteins CD33, CD68, CD69 or CD147 with S100A8 or S100A9, which would influence the release of proinflammatory molecules.

## Discussion

Identifying receptors and signaling pathways which are targeted and induced by endogenous inflammatory molecules like DAMPs is crucial for mechanistically understanding our immune system. S100A8/A9 is not only part of this molecular family but is among the most abundant ones in various inflammatory conditions (6). However, it is not sufficiently clarified which signaling pathways are activated by this DAMP and which receptors contribute to the signal transduction. A variety of receptors involved in the S100A8- and S100A9-mediated cellular response, most prominently TLR4, have been identified *via* various *in vitro* and *in vivo* experiments (7, 14–19).

Both the S100A8 and S100A9 homodimers as well as S100A8/S100A9 heterodimers induce the expression and release of TNF-α in monocytes *via* the TLR4-MD2-CD14 complex, mediated by MyD88 and NF-kB. The S100A8/S100A9 tetramer, which forms in a calcium-dependent manner, cannot act *via* this pathway due to the lack of accessibility to the TLR4 binding site, providing an autoregulatory mechanism to prevent an overshooting immune response (7).

In addition to the receptors CD33, CD68, CD69 and CD147 that were examined in this study, several other cell surface proteins have been associated with S100A8 or S100A9, including RAGE, CD36, or CD85j (39–41). Analysis of ER-Hoxb8-derived monocytes/macrophages obtained from RAGE knockout mice showed no restriction of the inflammatory activation of these cells in response to S100A8 stimulation (15). Similarly, transfection studies overexpressing RAGE excluded a relevant binding of S100A8 and S100A9. The same was true for overexpression of CD36 (42). We did not include CD85j in this study since a murine ortholog is not known and did not include RAGE a second time due to that these data are already published.

In the present study we could not detect any relevant interactions of S100A8 and S100A9 with CD33, CD68, CD69 or CD147 in our stimulation experiments on ER-Hoxb8-derived macrophages. Although some knockouts of these receptors induced minor differences in their MFI shifts, percentages of positive cells for the major monocyte differentiation markers Ly6C, F4/80 and CD11b showed no or only very minor differences (max. 90% versus 99% positive cells for Ly6C). The expression pattern differed more strongly only in CD68-KO cells for Ly6G and generally for CD11c which are no classical differentiation markers for the monocyte lineage. Thus, none of the knockouts induced a significant differentiation defect, especially none which could explain a false negative result of a specific receptor deletion. In addition, we controlled our evaluation considering the ratio of S100-induced cytokines in relation to stimulation with LPS to minimize even potential influences of minor alterations in differentiation. We could exclude significant differences between receptor-knockout and WT cells regarding inflammatory activation of ER-Hoxb8-derived monocytes and the cells reacted adequately to LPS stimulation and, more importantly, our S100 proteins showed the same pattern of activation.

CD69 is involved in the differentiation of regulatory T cells. *Via* immunoprecipitation and mass spectrometry, the S100A8/S100A9 complex was discovered by Lin et al. to be a ligand for this receptor. *Via* various *in vitro* assays, interactions were shown suggesting that STAT3 is downregulated by the binding of the complex to CD69, thereby directing the differentiation of regulatory T cells. These experiments were performed on human peripheral blood mononuclear cells and have not been further evaluated to date (18). Recent studies assign a different significance to the interaction between the S100 proteins and CD69, which relates to the S100A8/S100A9 tetramer. The S100A8/A9 tetramerization, which is induced in a calcium-dependent manner, has been characterized as an inhibitory mechanism of TLR4 stimulation. Recent findings show that the tetramer is instead antagonistically modulatory active and contributes to a resting state of leukocytes. Direct binding experiments have shown that the S100A8/A9 tetramer, but not the dimer, binds to CD69. The regulatory function of the tetramer could be demonstrated *in vitro* as well as *in vivo* in models of cutaneous granuloma and irritant contact dermatitis (30). A direct effect of S100-dimers on CD69 is now excluded by our study.

EMMPRIN was first described in the context of S100 proteins when S100A9 was discovered to be a promoter of melanoma cell migration (17). S100/EMMPRIN interaction has also been shown on keratinocytes, in this case with a particular focus on S100A8. Sakaguchi et al. showed that stimulation of keratinocytes with S100A8 activates keratinocyte proliferation *via* the receptor neuroplastin ß, which dimerizes with EMMPRIN (43). The discovery of S100A9 as an EMMPRIN ligand (17) already led to the investigation of a possible pro-inflammatory effect of S100A9 *via* EMMPRIN on monocytes/macrophages. Alexaki et al. used neutralizing antibodies against EMMPRIN to investigate the modulated migration of human monocytes. Migration could be induced by S100A9 and blocked again by the addition of an anti-EMMPRIN antibody. However, an anti-RAGE antibody achieved the same reduction and a corresponding TLR4 antibody showed no effect. Murine bone marrow-derived macrophages were also investigated in this study with regard to their cytokine and chemokine release. An upregulation of IL-6 and CXCL1 was observed under S100A9 stimulation, which was partially blocked by anti-TLR4 antibodies. Antibodies against EMMPRIN showed no effect (44). However, the efficacy of the antibodies used was not controlled within this experimental setup. Our knockout experiments now confirm the dominant role of TLR4 and exclude a significant involvement of EMMPRIN for S100-mdiated activation of macrophages with high specificity.

A single study by Okada et al. shows a possible proinflammatory interaction between CD68 and S100A8 and S100A9 in rat macrophages. First, direct binding of recombinant S100A8 or recombinant S100A9 to recombinant CD68 was shown by ELISA and affinity chromatography. For subsequent stimulation experiments, both an anti-CD68 antibody and corresponding siRNA were used instead of a knockout cell model. However, data presented in this study were somehow contradictory. In the siRNA stimulation experiments, clear differences between S100A8 and S100A9 were observed. However, the data obtained *via* antibody blockade differed substantially from those obtained from the corresponding gene silencing method. It is also notable that antibody-based TLR4 blockade partly resulted in the highest TNF-α and IL-6 expression under S100A9 stimulation evaluated by rtPCR (19). We can now exclude a relevant role of CD68 in S100-mediated inflammatory activation of macrophages.

The inhibitory function of CD33 induced by S100A9 in the context of myelodysplastic syndromes has been demonstrated by several studies. In 2013, S100A9 and CD33 were identified as a functional ligand/receptor pair that induces secretion of suppressive cytokines and accumulation of myeloid-derived suppressor cells *via* the ITIM domain of CD33. Binding and specificity were demonstrated *via* a transfection cell culture model, sandwich ELISA, immunoprecipitation (16), and in other studies by the use of a CD33-IgG_1_ fusion protein (a high-affinity S100A9 decoy receptor) (45) and *via* an Fc-engineered CD33-antibody (46). Corresponding knockout experiments or *in vivo* interaction studies of S100A9 and CD33 have not yet been performed, as has no potential activating signaling pathway been considered to date. In murine interaction studies, it might be a problem that murine CD33 lacks an intracellular ITIM domain, which has been described as crucial for the inflammatory signal transduction of CD33 (22, 47).

The monocytic ER-Hoxb8 cell system is superior to malignant cell lines and cell lines with artificial protein expression as it retains physiological properties and protein expression patterns of primary monocytes, thus establishing itself as a reliable tool in basic immunology research. ER-Hoxb8 cells are conditionally immortalized murine progenitor cells that express Hoxb8, a critical transcription factor that inhibits cellular differentiation, in an estrogen-dependent manner. Thus, differentiation can be initiated *via* estrogen withdrawal in a controlled process and also be further modified by the type of growth factor used. Differentiation into macrophages *via* GM-CSF has been described as functional and efficient in several studies (30, 37, 38, 48).

Equally modern and in the proliferative state of ER-Hoxb8 cells very efficient is the CRISPR/Cas9 gene editing system, which can be used to generate complete knockout cell lines even without the availability of appropriate knockout mouse strains (38). Applying the combination of these two methods guarantees a high level of specificity and biological relevance for the biology of monocytes/macrophages. With this approach we could exclude any significant functional relevance of interactions of S100A8 and S100A9 with the receptors CD33, CD68, CD69, and CD147 for the inflammatory activation of these cells. TLR4 has been discovered previously as a functional S100A8/S100A9 receptor on murine ER-Hoxb8 cells and therefore served as an appropriate control in the performed experiments (15).

Our approach may have some limitations. Since it is not yet known whether and to what extent the receptors investigated influence the overall survival and differentiation of the cells in the investigated cell system, the cells used were examined regarding their differentiation behavior prior to the final analysis. Slight differences were found here, which, however, had no significant consequences for the expression of any receptor analyzed in our study. These differences may explain variations in the absolute levels of cytokines secreted by the respective cell lines described above, but comparison with the response of wildtype and TLR4 knockout cells revealed a clear pattern. To characterize the cellular response of the cell lines studied we selected TNF-α and IL-6 for our analysis since both were strongly induced in wildtype ER-Hoxb8-derived macrophages and almost completely abrogated in TLR4 knockout cells in our setting. Furthermore, the relevance of the feedback between TNF-α and S100-proteins has been confirmed for inflammatory processes like arthritis and psoriasis (7) and IL-6 is the top cytokine induced by S100A8/S100A9 in myeloid cells as shown in a genome wide transcriptome analysis (15). Binding of S100A8/A9 to target cells may be additionally modified by glycosylation and glycan modification, as some S100 receptor/ligand interactions have been described as dependent on receptor glycosylation with sialic acid and S100A8/A9 has been shown to bind various glycosaminoglycans with high affinity (18, 42). Due to our approach we cannot draw conclusions regarding functions of the receptors addressed in this paper in other cellular systems, like tumor cells, or other functional states of myeloid cells like myeloid-derived suppressor cells in tumor environment or during myeloid dysplastic syndromes. As shown earlier by our group deletion of RAGE has no effect on S100-induced activation of murine monocytes but has been described to mediate S100-effect during colitis associated carcinogenesis (41). However, we focused our investigations on ER-Hoxb8 cells because the genetically manipulation of these cells occur in sufficient quantities without affecting viability or inducing activation of these cells. Additionally ER-Hoxb8-derived monocytes are comparable to primary cells and enable the analysis of inflammatory mechanisms in an easy und successful manner.

Overall, however, the results of this study support the assertion that TLR4 should still be considered the receptor with the highest relevance in the inflammatory response of macrophages to S100A8/S100A9 stimulation and that this response consists predominantly of the secretion of IL-6 and TNF-α.

## Data availability statement

The original contributions presented in the study are included in the article/**Supplementary Material**. Further inquiries can be directed to the corresponding author.

## Author contributions

AM: Conceptualization, Formal analysis, Investigation, Writing - original draft, Writing - review and editing. S-LJ-S: Methodology. TV: Funding acquisition, Project administration. JR: Conceptualization, Funding acquisition, Project administration, Supervision, Writing - review and editing. OF: Conceptualization, Investigation, Project administration, Supervision, Writing - review and editing, SG: Formal analysis. All authors contributed to the article and approved the submitted version. 

## Supplementary material

Supplementary Figure 1 Relative cytokine secretion upon S100A8 and S100A9 stimulation. CD33, CD68, CD69, CD147 and TLR4 knockout ER-Hoxb8 precursor cells, as well as WT ER-Hoxb8 precursor cells were differentiated into macrophages and subsequently stimulated with LPS, S100A8 homodimer, S100A9 homodimer or incubated without stimulus (Ctrl). TNF-α (A) and IL-6 (B) protein levels in cell culture supernatants were quantified using LegendPlex™ assay (n = 3). Concentrations below the limit of quantification were set as LLOQ/2. Fold changes of absolute cytokine values under S100 stimulation were calculated for each run with respect to mean cytokine values under LPS stimulation. Values are the means ± SEM. NS = not significant, by two-way ANOVA and Dunnett’s test in comparison to WT.

## Glossary

**Table:**

ANOVA	Analysis of Variance
Cas9	CRISPR-associated
CD	Cluster of Differentiation
CRISPR	Clustered Regularly Interspaced Short Palindromic Repeats
DAMP	Damage-associated molecular pattern
ELISA	Enzyme-linked Immunosorbent Assay
EMMPRIN	Extracellular Matrix Metalloproteinase Inducer
ER	Estrogen-regulated
Gal	Galectin
GM-CSF	Granulocyte-macrophage colony-stimulating factor
gRNA	guideRNA
HEK	Human embryonic kidney
Ig	Immunoglobulin
IL	Interleukin
ITIM	Immunoreceptor tyrosine-based inhibitory motif
Jak	Janus kinase
KO	Knockout
lamp	Lysosomal-associated membrane protein
LLOQ	Lower limit of quantification
LPS	Lipopolysaccharide
MAPK	Mitogen activated protein kinase
MFI	Mean fluorescent intensity
MMP	Matrix metalloproteinase
Mrp	Myeloid-related protein
MyD88	Myeloid differentiation primary response 88
NF-κB	Nuclear factor kappa-light-chain-enhancer of activated B cells
NS	Not significant
oxLDL	Oxidated Low-Density Lipoprotein
PAM	Protospacer adjacent motif
PAMP	Pathogen-associated molecular pattern
PCR	Polymerase chain reaction
PRR	Pattern recognition receptor
RAGE	Receptor for advanced glycation endproducts
SEM	Standard error of the mean
SHP	Src homology 2 domain-containing protein tyrosine phosphatase
Siglec	Sialic acid-binding immunoglobulin-type lectin
siRNA	Small interfering RNA
STAT	Signal transducer and activator of transcription
TGF	Transforming growth factor
TLR	Toll-like receptor
TNF	Tumor necrosis factor
WT	Wild-type
