# Supplementary figures and images for: The roles of toll-like receptor 4, CD33, CD68, CD69, or CD147/EMMPRIN for monocyte activation by the DAMP S100A8/S100A9
Source: Front Immunol. 2023 Mar 28;14:1110185. doi: 10.3389/fimmu.2023.1110185 (PMC10086345; doi:10.3389/fimmu.2023.1110185)

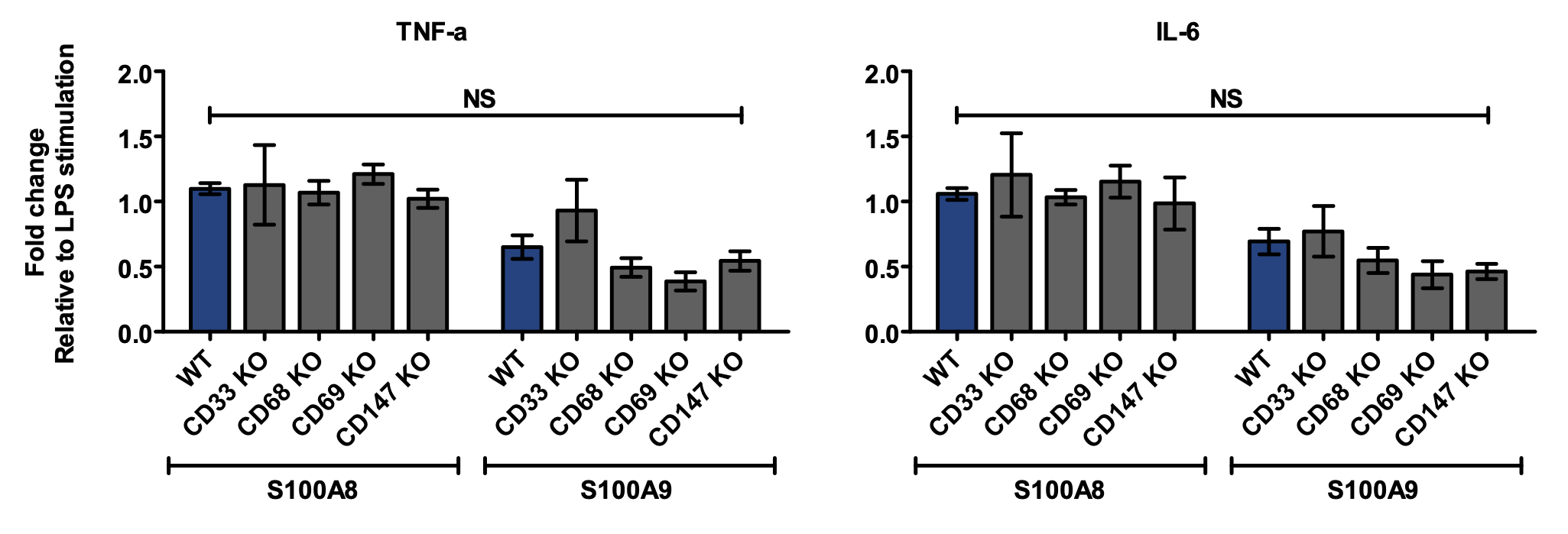

Supplement: Supplementary Figure 1 — Relative cytokine secretion upon S100A8 and S100A9 stimulation. CD33, CD68, CD69, CD147 and TLR4 knockout ER-Hoxb8 precursor cells, as well as WT ER-Hoxb8 precursor cells were differentiated into macrophages and subsequently stimulated with LPS, S100A8 homodimer, S100A9 homodimer or incubated without stimulus (Ctrl). TNF-α (A) and IL-6 (B) protein levels in cell culture supernatants were quantified using LegendPlex™ assay (n = 3). Concentrations below the limit of quantification were set as LLOQ/2. Fold changes of absolute cytokine values under S100 stimulation were calculated for each run with respect to mean cytokine values under LPS stimulation. Values are the means ± SEM. NS = not significant, by two-way ANOVA and Dunnett’s test in comparison to WT. [file Image_1.tiff]
