# Supplementary material for: The roles of toll-like receptor 4, CD33, CD68, CD69, or CD147/EMMPRIN for monocyte activation by the DAMP S100A8/S100A9
Source: Front Immunol. 2023 Mar 28;14:1110185. doi: 10.3389/fimmu.2023.1110185 (PMC10086345; doi:10.3389/fimmu.2023.1110185)
Supplement: Supplementary file 2 [file Table_1.docx]

Target FW RV

CD33 CACCGGGCAGATTCCAACTCCTTG AAACCAAGGAGTTGGAATCTGCCC

CD68 CACCGTAGGTGTCATCGTGAAGGA AAACTCCTTCACGATGACACCTAC

CD69 CACCGGTGAACTGGAACATTGGAT AAACATCCAATGTTCCAGTTCACC

CD147 CACCGCCTACCCTCCTATTACAGAT AAACATCTGTAATAGGAGGGTAGGC

TLR4 CACCGAATCCTGAGCAAACAGCAG AAACCTGCTGTTTGCTCAGGATTC

**Table S1: gRNA sequences (5’ – 3’) for cloning into lentiGuide-puro**

Target FW RV

CD33 AGGGGTCAGTCTCCATGAAG AGGTCACATGCAGTGACAG

CD68 ACCAATCTCTTACTGAAAGCCCA TTATGAGTGACAGTTGTGGGTCC

CD69 CACCGGTGAACTGGAACATTGGAT AAACATCCAATGTTCCAGTTCACC

CD147 TCAAGGTCGGAAAGAAATCAG TTAGCCTCACAGCCCTTAC

TLR4 CACCGAATCCTGAGCAAACAGCAG AAACCTGCTGTTTGCTCAGGATTC

**Table S2: PCR primers (5’ – 3’) flanking gRNA target regions**

Antibody Manufacturer Cat.

APC anti-mouse CD284 Biolegend 145405

APC anti-mouse CD68 Biolegend 137008

APC anti-mouse CD69 Biolegend 104513

APC anti-mouse F4/80 Biolegend 123116

APC anti-mouse Ly-6G Biolegend 127614

APC Armenian Hamster IgG Isotype Ctrl Biolegend 400911

APC Rat IgG2a, κ Isotype Ctrl Biolegend 400512

CD33 Monoclonal Antibody, PE ThermoFisher Scientific 12-0331-80

Pacific Blue™ anti-mouse/human CD11b Biolegend 101224

Pacific Blue™ Rat IgG2b, κ Isotype Ctrl Biolegend 400627

PE anti-mouse CD11c Biolegend 117308

PE anti-mouse CD147 Biolegend 123707

PE anti-mouse Ly-6C Biolegend 128008

PE Armenian Hamster IgG Isotype Ctrl Biolegend 400908

PE Rat IgG1, κ Isotype Ctrl Biolegend 400408

PE Rat IgG2c, κ Isotype Ctrl Biolegend 400707

**Table S3: Antibodies and isotype controls for flow cytometry**
